# Memory in Psychiatric Disorders: A Review

**DOI:** 10.3390/life15121926

**Published:** 2025-12-16

**Authors:** Riccardo Gurrieri, Matteo Gambini, Gerardo Russomanno, Federico Mucci, Manuel Glauco Carbone, Giorgia Sità, Elena Pescini, Sibilla Stagi, Anna Chiara Casucci, Diletta Mastrogiacomo, Francesca Bressan, Donatella Marazziti

**Affiliations:** 1 Department of Clinical and Experimental Medicine, Section of Psychiatry, University of Pisa, 56100 Pisa, Italy; riccardogurrieri4@gmail.com (R.G.); gambinimatteo1996@gmail.com (M.G.); giorgiasita@gmail.com (G.S.); stagisibilla@gmail.com (S.S.);; 2 Department of Psychiatry, Lucca Zone, Azienda USL Toscana Nord Ovest, 55100 Lucca, Italy; 3 Department of Medicine and Surgery, Division of Psychiatry, University of Insubria, 22100 Varese, Italy; 4 Centro Psicologia Parioli, 00197 Roma, Italy

**Keywords:** memory, memory dysfunction, working memory, episodic memory, autobiographical memory, psychiatric disorders, targeted interventions

## Abstract

Memory constitutes a fundamental cognitive domain, and converging evidence suggests that its dysfunction represents a prominent, though not exclusive, transdiagnostic dimension across major psychiatric disorders. This review aimed to integrate neurobiological, cognitive, and clinical evidence on domain-specific memory impairments in mood, anxiety, obsessive–compulsive, post-traumatic stress, and psychotic disorders. A comprehensive search was conducted on PubMed, Scopus, and Web of Science up to November 2025 for peer-reviewed studies examining short-term, working, long-term, episodic, semantic, and prospective memory, prioritizing both landmark and recent contributions. Two recurrent transdiagnostic patterns emerged: (i) consistent impairments in working-memory control, and (ii) reduced episodic/autobiographical specificity, while procedural memory appeared relatively preserved. Disorder-specific profiles include overgeneral autobiographical memory in major depression, enduring working and episodic deficits in bipolar disorder, variable impairments in anxiety disorders, functional rather than structural memory inefficiencies in obsessive–compulsive disorder, broad mnemonic disorganization in post-traumatic stress disorder, and pervasive working and episodic deficits in schizophrenia and related psychoses. Across conditions, converging neurobiological data implicate fronto-hippocampal dysconnectivity, altered plasticity, and impaired consolidation processes. Unlike previous reviews, this work syntetisizes evidence across multiple memory systems and across major psychiatric categories, linking neurobiological mechanisms with cognitive and clinical manifestations to support a dimensional, transdiagnostic interpretation of memory dysfunction. These findings could suggest that memory dysfunction represents a recurrent and clinically relevant dimension across psychiatric conditions, warranting further mechanistic and longitudinal investigation.

## 1. Introduction

Memory is the ability to remember and is essential for human existence, enabling individuals to reconstruct the past from the present, while ensuring the continuity of knowledge [1,2]. It is a dynamic and complex system that allows individuals to encode, store, and retrieve past experiences, facilitating the navigation of current and future life situations [3]. Rather than a static or passive archive, memory is an active process that continuously constructs representations of the world, shaping our interactions and decisions.

Memory processing involves distinct functional processes and systems in different brain regions [4]. Encoding refers to the initial processing of stimuli, while consolidation transforms them into stable memory traces [5,6]. Storage involves maintaining learned information over time, and retrieval is the process of bringing stored information into conscious awareness [7].

Memory is typically distinguished into sensory, short-term (STM), working (WM), and long-term (LTM). Sensory memory rapidly processes sensory input, identifying and selecting the most relevant stimuli [8]. Short-term memory temporarily retains information essential for cognitive tasks, while long-term memory stores acquired knowledge and experiences throughout one’s life [9]. Working memory, distinguished in iconic memory retaining visual stimuli and echoic memory holding auditory information, enables the maintenance, integration, and manipulation of information for specific cognitive purposes [10]. For these characteristics, it is fundamental for decision making [11]. This system includes components such as the visuospatial sketchpad, articulatory loop, and episodic buffer, with the central executive component overseeing their coordination [12]. Long-term memory is categorized into declarative and non-declarative systems. Declarative memory, which includes episodic and semantic memory, is conscious and can be verbalized. Non-declarative memory, including procedural memory and priming, operates outside conscious awareness and is implicit in both encoding and retrieval [10].

Despite numerous disorder-specific reviews, no prior work has integrated findings across major psychiatric conditions and across multiple memory systems within a unified framework. However, existing literature remains fragmented. Reviews typically focus on single disorders or isolated memory domains, and few synthesize neurobiological, cognitive, and clinical findings across psychiatric categories. A transdiagnostic approach is increasingly warranted, as emerging evidence suggests that specific aspects of memory, particularly working-memory control and episodic/autobiographical specificity, are disrupted in multiple disorders and linked to shared neural circuits. Situating these alterations within a dimensional framework allows us to move beyond categorical boundaries and examine common mechanisms alongside disorder-specific profiles. This narrative review aims to synthesize evidence on the neurobiological underpinnings of the memory processes and to critically examine domain-specific memory alterations across major psychiatric disorders (mood, anxiety, obsessive–compulsive disorder [OCD], post-traumatic stress disorder [PTSD], and psychotic disorders). Accordingly, this review addresses four guiding questions:

(1) Which memory domains are most consistently affected across mood, anxiety, obsessive–compulsive, trauma-related, and psychotic disorders?
(2) What neurobiological and network-level mechanisms may account for shared or divergent patterns of impairment?
(3) How do disorder-specific profiles relate to transdiagnostic trends?
(4) What are the clinical implications for treatment, and cognitive intervention?

We mapped transdiagnostic regularities and disorder-specific profiles, linked cognitive findings to neural circuits and mechanisms (from systems- to synaptic-level plasticity), and discussed clinical implications and therapeutic strategies to target memory dysfunction.

## 2. Methods

This review was conducted as a comprehensive synthesis of the existing literature on memory functions and deficits across major psychiatric disorders. Relevant articles were identified through searches of PubMed, Scopus, and Web of Science up to November 2025, using combinations of the following keywords: “Memory,” “Working Memory,” “Short-term Memory,” “Long-term Memory,” “Episodic Memory,” “Prospective Memory,” “Psychiatric Disorders,” “Mood Disorders,” “Anxiety Disorders,” “OCD,” “Psychotic Disorders,” “PTSD.”

Inclusion criteria were: (i) peer-reviewed original research articles or reviews; (ii) studies involving human participants with a diagnosis of major psychiatric disorders, and (iii) studies reporting cognitive, neuropsychological, or neurobiological findings related to memory functions. Exclusion criteria were: (i) studies focusing exclusively on animal models without translational discussion; and (ii) articles not published in English. Selection criteria included methodological rigor, sample size, the availability of convergent behavioral and neurobiological evidence, and the relevance and significance of transdiagnostic mechanisms.

First, we prioritized investigations that explicitly linked at least one well-defined memory domain (e.g., working, episodic, autobiographical, semantic, or prospective memory) with major psychiatric diagnoses (mood, anxiety, obsessive–compulsive, trauma-related, and psychotic disorders), rather than examining non-clinical samples or undifferentiated cognitive composites. Second, we favored studies that integrated cognitive or neuropsychological data with neurobiological, neuroimaging, or clinically relevant outcomes (e.g., functional impairment, symptom dimensions), in order to highlight mechanistic and transdiagnostic patterns rather than isolated task effects. Third, when multiple reports addressed the same question, we gave precedence to meta-analyses, large-sample or longitudinal studies, and those providing direct comparisons across disorders or illness stages (e.g., first-episode vs. chronic, acute vs. remitted). Finally, we prioritized evidence that spoke to cross-diagnostic regularities in working-memory control, episodic/autobiographical specificity, and the relative preservation of procedural learning, while still describing disorder-specific profiles where data were sufficiently consistent.

The synthesis was conducted qualitatively. When multiple publications originated from the same cohort, the most comprehensive or methodologically robust report was retained to avoid redundancy. Heterogeneity in diagnostic criteria, illness phase, comorbidity, and assessment tools was acknowledged and incorporated into the qualitative interpretation. Minimal methodological adequacy was ensured by excluding studies lacking standardized cognitive measures or clear diagnostic characterization.

Given the narrative design, no formal quantitative synthesis or risk-of-bias assessment was performed. Instead, the evidence was critically evaluated to highlight consistent findings, discrepancies, and emerging trends across disorders, with particular attention to neurobiological correlates and their clinical implications.

## 3. Memory as a Transdiagnostic Dimension: Neurobiology and Disorder-Specific Profiles

### 3.1. Neurobiology of Memory

The first significant clues to the neurobiological basis of memory in humans came from the study of the famous patient H.M., who in 1953 underwent neurosurgery to treat severe, intractable temporal lobe epilepsy that had afflicted him since childhood [13,14]. The procedure involved the bilateral removal of the hippocampus, amygdala, and part of the multimodal associative cortex of the temporal lobe. Following the operation, H.M. exhibited profound deficits in the ability to form new memories, while retaining good recall of events that had occurred before surgery. Specifically, he was unable to establish new semantic and episodic long-term memories, although his working memory, lasting only a few seconds, remained intact, as did his procedural memory [13,14]. These findings highlighted the critical role of the medial temporal lobe, particularly the hippocampus, in memory formation. Supporting evidence came from another patient, R.B., who developed qualitatively similar, although milder, memory deficits after selective damage to the CA1 pyramidal cells of the hippocampus [15]. Together, these cases demonstrated that the medial temporal lobe is central to the encoding, storage, consolidation, and recall of declarative memory. Moreover, the deficits observed in human patients can be replicated in experimental animals through targeted lesions in the hippocampus and in other brain areas, making memory a diffuse system in the brain [15,16].

In the following sections, we will briefly summarize the characteristics of each type of memory, together with the main findings on their neural circuits, as well as the molecular basis of memory formation and consolidation.

Short-term memory (STM) refers to the temporary maintenance of limited information over brief periods of time. According to the model of Atkinson and Shiffrin (1968) [9], following an earlier work [17], STM should be considered a storehouse receiving inputs from sensory registers and LTM, while also contributing to reasoning and the generation of new inferences. This model highlighted the role of short-term storage as a precursor to long-term retention and as a core component of the executive system responsible for coordinating complex cognitive operations. However, subsequent findings challenged this model, particularly the notion that a longer retention in STM increases the probability of transfer to long-term storage [18,19]. These criticisms prompted the emergence of alternative hypotheses, including that of Craik and Lockhart’s levels of processing framework, of Cowan’s embedded-processes models, and of Goldman-Rakic’s neurobiological perspectives [20,21]. However, Baddeley and Hitch’s WM model became the most widely accepted, as it reconceptualized STM as a multi-component system for both storage and active manipulation of information rather than a unitary temporary store [22].

Working memory is a distributed cognitive system primarily supported by dynamic interactions between the prefrontal (PFC) and the posterior parietal cortex (PPC) [23]. Each WM subcomponent mentioned above, the central executive, phonological loop, visuospatial sketchpad, and episodic buffer [12,22], involves distinct, albeit interconnected neural substrates. A large-scale network encompassing the cingulo-opercular and fronto-parietal systems modulates the central executive, coordinates attention, and integrates activity across subsystems [24,25]. The phonological loop relies on left-lateralized perisylvian regions, including Broca’s and Wernicke’s areas, which support rehearsal and storage of verbal information [26,27]. The visuospatial sketchpad engages right-lateralized occipital, parietal, and prefrontal regions in maintaining and manipulating visuospatial representations [28,29]. The episodic buffer integrates multimodal information and interfaces WM with LTM, although it is not confined to a single anatomical locus [30]. Recent theoretical developments, that attentional allocation to internal representations may underpin short-term retention [31]. These models emphasize the role of persistent neural activity, particularly in the PFC, supported by synaptic and oscillatory mechanisms that sustain task-relevant representations across distributed cortical and subcortical regions [32]. Moreover, evidence highlights how progressively rostral areas support increasingly abstract forms of cognitive control, enabling flexible goal-directed behavior [33]. Overall, converging evidence suggests that WM would emerge from the coordinated activity of distributed neural systems that manipulate information according to task demands [34].

Long-term memory can be broadly categorized into declarative (explicit) and non-declarative (implicit) systems. Declarative memory depends critically on medial temporal lobe structures, particularly the hippocampus, and supports the conscious recollection of facts and events [15,16,35]. Two major subtypes of declarative memory can be identified: the episodic memory, which refers to the recollection of personally experienced events situated in a spatial and temporal context, and the semantic memory, which encodes factual and conceptual knowledge independent from specific episodes [36]. Retrieval of episodic memory relies on coordinated activity between the hippocampus, ventral parietal cortex (VPC), and PFC, which jointly support the recovery of contextual details such as the spatial and temporal features of past events [37,38]. Within this network, the medial PFC (mPFC) has been implicated in encoding processes, while the lateral PFC (lPFC) contributes to retrieval and the integration of multimodal inputs [39]. The amygdala further modulates episodic memory by enhancing the encoding and recall of emotionally salient experiences through its interaction with hippocampal circuitry [40]. Since the 1990s, positron emission tomography (PET) and functional magnetic resonance imaging (fMRI) studies-consistently demonstrated activation of the hippocampal formation and medial temporal lobe (MTL) structures during episodic memory tasks, particularly when encoding stimuli that are subsequently remembered compared to those that are forgotten [41,42,43]. However, such findings must be interpreted with caution, as the variability of MTL subregions complicates the assessment [44]. Event-related fMRI designs have advanced this field by revealing differential contributions of MTL to encoding and retrieval, although results remain controversial. Some studies reported rostral hippocampal activation during encoding and caudal activation during retrieval [45], while others found the opposite [46], or dissociations across subfields [47]. Moreover, perirhinal and rostral hippocampal regions are associated with faces, caudal regions with objects, and posterior parahippocampal cortex with spatial contexts such as indoor and outdoor scenes [48]. Evidence also suggests that the hippocampal formation underlies associative memory and recollection, while perirhinal and entorhinal cortices contribute to familiarity-based judgments [49]. Further data highlighted the role of hippocampal subfields in pattern separation and completion [50] and in supporting inferential reasoning processes such as transitive inference, whereby previously learned associations are flexibly recombined to generate novel knowledge [51]. Taken together, neuroimaging and lesion evidence underscore the hippocampal formation as a central hub for encoding, retrieval, and the flexible integration of episodic details, in close interaction with prefrontal and parietal cortex as well as amygdala.

Semantic memory relies on a distributed network encompassing the inferior parietal lobe, lateral and anterior temporal cortices, and multimodal association areas supporting the conceptual representations essential for language, object recognition, social cognition, and the capacity to imagine future scenarios [36,52]. Neuroimaging studies highlight the role of anterior, ventral and left inferior frontal gyrus (IFG) in tasks selection among competing semantic representations, while the dorsomedial prefrontal cortex (dmPFC) appears to support self-initiated retrieval processes and the integration of affective and cognitive control systems [53]. Lesion evidence confirms that damage to the superior frontal gyrus disrupts self-guided semantic retrieval [54]. Although the temporal pole (TP) has been proposed as a central hub for amodal semantic representations, converging evidence suggests that semantic processing depends on broader temporal and parietal areas, including the angular gyrus (AG), which is sensitive to lexical and conceptual richness [36,52].

To summarize, available data suggest that semantic memory would emerge from the intertwining among distributed cortical regions, with frontal areas contributing to control and selection, temporal areas to conceptual representation, and parietal areas to the integration of events within spatial and temporal frameworks.

Non-declarative memory refers to unconscious learning processes that operate independently from awareness, including skills and habits (procedural memory), priming, conditioning, and associative or non-associative learning [15,55]. This type of memory is less dependent on the hippocampus and relies on specialized, domain-specific networks. Procedural memory involves circuits spanning the motor cortex, striatum, cerebellum, and limbic system, supporting the gradual acquisition of motor and perceptual skills [56]. Priming mechanisms are associated with reduced neural activation (“repetition suppression”) in sensory and prefrontal regions, enabling more efficient processing of previously encountered stimuli [53,57]. Moreover, recent research highlighted the role of associative memory cells, which emerge from the co-activation of interconnected regions and contribute to the integration and retrieval of related experiences [58,59]. Therefore, non-declarative memory reflects a heterogeneous set of systems with distinct neural substrates, each specialized in supporting adaptive, automatic behaviors shaped by experience.

Both declarative and non-declarative memory systems rely on consolidation processes that stabilize initially fragile representations into durable traces. Memory consolidation occurs at two interdependent levels. Synaptic (or cellular) consolidation involves activity-dependent changes at the neuronal level, primarily through long-term potentiation (LTP) and long-term depression (LTD) in hippocampal circuits, which rapidly stabilize the memory trace following encoding [60]. In parallel, systems consolidation entails a slower redistribution of memory representations from hippocampal–medial temporal lobe structures to neocortical networks, thereby promoting long-term stability and resistance to interference [7,61]. This process aligns with Hebbian principles of learning, whereby repeated co-activation of neurons enhances their likelihood of firing together, thus reinforcing long-term associations [62]. Through these mechanisms, memory traces, whether explicit or implicit, are gradually transformed into enduring, accessible knowledge structures that shape cognition and behavior across the lifespan.

Synaptic plasticity, particularly LTP and LTD, represents a fundamental mechanism underlying memory stabilization by modifying synaptic strength [63]. Long-term potentiation, characterized by a persistent increase in synaptic efficacy following high-frequency stimulation, is most often mediated by the N-methyl-D-aspartate (NMDA) receptor-dependent processes in which glutamate release activates the α-amino-3-hydroxy-5-methyl-4-isoxazolepropionic acid (AMPA) and NMDA receptors, triggering calcium influx and downstream cascades that enhance AMPA receptor conductance and promote receptor insertion in the postsynaptic membrane [64]. Long-term potentiation at the basis of memory consolidation, unfolds in two phases: an early phase (E-LTP), which is protein-synthesis independent and transient, and a late phase (L-LTP), which requires gene transcription and protein synthesis mediated by the cAMP response element-binding protein (CREB) and the CCAAT/enhancer-binding protein (C/EBP) activation, thus sustaining memory traces for extended periods [65,66]. The synaptic tagging and capture hypothesis further explains how plasticity-related proteins (PRPs) generated through strong stimulation at neighboring synapses may stabilize weakly induced LTP can be stabilized by [67,68], a process in which calcium-permeable AMPA receptors (CP-AMPARs) and dopaminergic signaling play key roles [69,70]. Three essential properties of LTP, associativity, synapse specificity, and cooperativity, support its role in encoding memories by selectively strengthening coactive neural connections while preserving input specificity [66]. Complementing this, LTD, a long-lasting decrease in synaptic efficacy, contributes to synaptic reorganization and has been implicated in processes such as spatial memory regulation [71]. Notably, it has been demonstrated that associative fear memories can be inactivated by LTD and reactivated by LTP, establishing a causal link between synaptic plasticity and memory storage [71]. This initial cellular consolidation is followed by systems consolidation, during which hippocampal-dependent memories are gradually re-organized across distributed cortical networks [72].

All together, these findings underscore synaptic plasticity as the cellular substrate of learning and memory, linking molecular cascades at the synapse to long-term systems-level stabilization of memory. Across different disorders; however, comparative interpretation remains limited by the substantial heterogeneity of memory paradigms. Tasks generally differ widely in modality, load, emotional salience, delay interval, and scoring procedures, reducing direct comparability between studies. This paper emphasizes patterns observed in convergent task types rather than isolated findings, without excluding them. This perspective enables us to examine both common patterns of dysfunction and disorder-specific features within a coherent conceptual model.

### 3.2. Memory in Mood Disorders

#### 3.2.1. Major Depressive Disorder

Major depressive disorder (MDD) is a highly prevalent psychiatric condition, associated with severe functional impairments, intense subjective distress, and an increased risk of suicide [73]. According to the World Health Organization, it affects millions of people every year and represents one of the leading causes of disability worldwide [73,74].

The most common symptoms include depressed mood and loss of pleasure or interest in usual activities, loss of interest in usual activities, unwarranted feelings of guilt or worthlessness, difficulty concentrating, recurrent thoughts of death, and changes in appetite, sleep, or energy levels. In many cases, the reported distress is disproportionate to external events or may arise in the absence of any identifiable cause [75,76].

Cognitive dysfunction, particularly memory deficits, is a key feature of the disorder is which significantly affects daily functioning and quality of life [77]. Episodic memory, enabling the recollection of autobiographical events in space and time, appears selectively impaired in MDD [78,79,80,81,82,83,84]. Patients often produce vague, generic memories when recalling personal events, a phenomenon known as overgeneral autobiographical memory (OGM) [79,81,85]. This pattern limits access to positive memories and interferes with emotional regulation [81,86].

From a neurobiological perspective, episodic and autobiographical memory impairments are supposed to reflect structural and functional anomalies in the hippocampus, amygdala, prefrontal cortex, and basal ganglia. Different data show that depressed patients frequently exhibit reduced hippocampal volume, impaired neurogenesis in the dentate gyrus, and amygdala hyperactivity [79,87]. In addition, altered connectivity between the hippocampus and prefrontal cortex would hinder the formation of positive memories and the suppression of negative ones, increasing relapse risk [87,88]. Interestingly, neuroimaging further shows hypoactivation of the hippocampus, DLPFC, and ACC during encoding and retrieval tasks [80,89]. Verbal episodic memory is also consistently impaired in depression, especially in major and mixed anxiety-depressive disorders [90], and it may act as a premorbid marker that may persist even after clinical remission [79,91].

Beyond EM, the WM deficits are robustly documented [92,93,94,95,96]. In n-back tasks, patients show reduced accuracy and slower responses under moderate load, reflecting psychomotor slowing and impaired executive control [97,98]. Compensatory hyperactivation in the DLPFC and ACC suggests greater cognitive effort is required to maintain performance [99,100]. Working memory dysfunction appears driven by reduced motivation, catastrophic responses to failure, attentional bias toward negative stimuli, and impaired cognitive inhibition [101].

Short-term memory is moderately impaired, especially in tasks involving delayed retention or high load, and its deficits are primarily attributed to impaired attentional and executive mechanisms [92,102]. Short-term memory’s impairments are less severe than those in WM and EM but still affect daily functioning [103]. Neuroimaging reveals hypoactivation of the DLPFC during STM tasks [104,105]. At neurophysiological level, depression is linked to accelerated decay of temporal memory and impaired motor preparation, reflected in premature saccades and altered inhibitory control [106].

In conclusion, MDD is a multidimensional disorder where EM, autobiographical, WM and STM deficits constitute a central element of psychopathology [77,78,79,80,82,84]. These impairments possibly stem from structural and functional alterations in memory-related circuits, reduced synaptic plasticity, impaired neurogenesis, and dysregulation of the HPA axis [107,108].

A few data in dysthymia, now called Persistent Depressive Disorder (PDD), a condition with less severe, albeit chronic and invalidating depressive symptoms [109], would suggest cognitive profiles analogous to MDD, with milder disruptions in EM and WM [110,111,112,113].

Despite the robust pattern of deficits in episodic and autobiographical memory, several studies using recognition-based paradigms or lists of emotionally neutral words have reported preserved accuracy, in contrast to the results obtained with free recall and autobiographical specificity tasks. This divergence likely reflects a task-dependent sensitivity; paradigms requiring self-referential processing consistently reveal deficits, while more structured tasks reduce the impact of affective bias. Similarly, variable results are observed in working memory, where n-back studies often reveal deficits while digit span performance is often normal, highlighting how workload and emotional salience critically influence results [80,83,84,87,89,99,100].

#### 3.2.2. Bipolar Disorders

Bipolar disorders (BDs), including type I (BDI) and type II (BDII), are chronic psychiatric conditions characterized by mood swings ranging from manic or hypomanic episodes to depressive episodes [109]. Beyond mood instability, the presence of cognitive deficits is an increasingly recognized feature of BD, which frequently affects EM (verbal and visuospatial), WM, verbal learning, processing speed, sustained attention, and sensorimotor skills [114,115,116]. These deficits are often more pronounced during acute episodes (manic, depressive, or mixed states), but may persist into euthymic phases [117,118]. This might indicate that cognitive dysfunction represents a core feature of the disorder rather than a transient symptom of mood episodes [116,119].

Similar impairments have been observed in first-degree relatives, suggesting a genetic or familial component [120]. Factors such as sleep disturbances and psychotropic medications may contribute, although deficits persist even in medicated euthymic patients, reinforcing their role as potential endophenotypes [118,121]. Again, some evidence suggests memory impairments are present from the first episode of BD, supporting the hypothesis of early neurocognitive vulnerability [121].

Neuroimaging studies show reduced activation in the DLPFC and ACC during n-back tasks, particularly under emotional load [122,123]. These alterations are consistent with the “Processing Efficiency Theory” [124] and the “Attentional Control Theory” [125], which postulate that anxiety disrupts attentional control [126]. Even in euthymic phases, BD patients exhibit differential responses to emotional stimuli, with greater amygdala and ACC activation for negative valence and enhanced connectivity for positive valence [116,123].

Direct comparisons of BDI vs. BDII are scanty. One study reported distinct activation patterns, with BDI showing more severe cognitive impairments, while BDII exhibited broader emotional-cognitive difficulties [127]. BDI tends to associate with psychotic symptoms, whereas BDII carries a higher risk of suicidal behavior [128,129]. Hospitalized BD-I patients perform worse cognitively than outpatients [130].

Besides WM, EM deficits, particularly verbal and autobiographical, are consistently observed, even in euthymia, pointing to stable neurocognitive vulnerability [131,132,133,134]. Persistent verbal EM impairments in BDI have been repeatedly documented [134,135], with neuroimaging studies confirming altered prefrontal, limbic, and occipital activity during EM tasks [131]. Autobiographical memory, central to identity and social functioning [136], is frequently overgeneralized and vague [137,138], while impairing self-awareness and relationships. An interesting finding concerns the influence of mood state during memory encoding. Memories formed during manic episodes are more difficult to retrieve than those formed during depressive or euthymic phases, probably because mania seems to interfere with the consolidation of personal memory [133].

Findings on STM are inconsistent. Some studies suggest preserved function in low-demand tasks, while others report reduced processing efficiency, particularly in BDI [139]. Overgeneralized autobiographical recall has also been linked to executive dysfunction and appears independent from current mood state [140,141].

In BD, cognitive impairment represents a core clinical feature rather than a secondary consequence of mood episodes. Deficits are most evident in WM and EM, often persisting into euthymia and observed even in first-degree relatives, suggesting a trait-like vulnerability. These alterations, strongly modulated by emotional states and underpinned by fronto-limbic dysfunction, highlight memory impairment as a potential endophenotypic marker of the disorder [123,133,136,142].

Evidence of episodic memory deficits is generally consistent across verbal learning tests, but less consistent across visuospatial paradigms, with several studies reporting intact performance on picture recall measures despite deficits in narrative or word list retention. This heterogeneity suggests differential vulnerability of memory subsystems depending on emotional load and executive demands. Similarly, while recurrent working memory deficits are evident under conditions of high cognitive load, simpler span tasks often produce contradictory results, indicating a dissociation between core maintenance and manipulation processes across bipolar disorder subtypes and clinical states [122,123,127,131,132,133,142].

### 3.3. Memory in Anxiety Disorders

Anxiety disorders are characterized by excessive fear and anxiety with autonomic arousal, maladaptive cognitive patterns (e.g., worry, threat bias), and avoidant behaviors, leading to clinically significant impairment in social, occupational, or relational functioning. Common manifestations include tachycardia, sweating, tremors, muscle tension, restlessness, difficulties in concentration and sleep regulation, hypervigilance, and reassurance seeking [143,144].

The most prevalent anxiety disorders include: Generalized Anxiety Disorder (GAD), Panic Disorder (PD), agoraphobia, Social Anxiety Disorder (SAD), separation anxiety disorder, selective mutism.

Beyond their core emotional symptoms, anxiety disorders are frequently associated with impairments in memory, largely mediated by disrupted attentional control and emotion regulation [145]. These deficits are often linked to disruptions in attentional control and emotional regulation, which compromise core memory processes such as encoding, consolidation, and retrieval [145,146]. The “Attentional Control Theory” [125] remains the dominant framework, while stating that anxiety may compromise executive components of WM (inhibition, shifting), bias attention toward threat, and deplete resources through worry and rumination [147,148]. Worry, a central cognitive feature of anxiety, further depletes attentional resources, reducing flexibility and accuracy [149]. These impairments are observed across age groups, even in pediatric samples, as anxious individuals show reduced accuracy and efficiency in verbal and visuospatial WM (VSWM) tasks, longer task durations, and increased effort [150,151]. High anxiety consistently correlates with reduced WM capacity [152,153], although some studies report preserved or enhanced WM in individuals with high trait social anxiety under no-distractor conditions [154]. At neurobiological level, spatial WM deficits may arise from competition between anxious arousal and cognitive processes for neural resources in the right prefrontal and posterior parietal cortices [153]. More broadly, reduced WM efficiency may reflect an inefficient allocation of resources between hemispheres within the DLPFC or an overall reduction in its functional capacity [155]. Youth with anxiety disorders also show increased amygdala VMPFC connectivity during threat processing and altered activation of the inferior temporal cortex during memory tasks, compared to healthy controls [156]. However, the literature presents mixed findings: while most studies confirm impairments in WM, prospective memory, and visuospatial tasks [157,158], others report no significant differences between anxious individuals and controls [159].

#### 3.3.1. Generalized Anxiety Disorder

The literature on GAD is quite controversial. A series of studies supports the presence of persistent WM impairments even during low-load tasks, suggesting that poor top-down regulation diverts attentional resources from goal-directed behavior to emotional control [160]. In line with these findings, another study reported reduced WM accuracy and slower reaction times in individuals with GAD under both safe and threatening conditions [155]. Furthermore, evidence shows that deficits in WM performance in GAD are negatively correlated with baseline worry and symptom severity measures [161].

These impairments have been hypothesized to reflect reduced PFC involvement as a trait feature of clinical anxiety, rather than a state-dependent response to threat [155]. Notably, findings highlight reduced activity and decreased white matter volume in the DLPFC [162]. More broadly, patients with GAD may exhibit impairments in the amygdala, posterior parietal regions, ACC, DLPFC, and VLPFC, particularly in the right hemisphere [163]. However, other studies failed to detect significant impairments in verbal or non-verbal memory when comparing individuals with GAD to healthy controls [164,165,166]. It has been suggested that, in the absence of threat or high cognitive load, individuals may engage compensatory strategies to maintain performance, a notion consistent with the “Attentional Control Theory” [125,165]. Similarly, studies in pediatric populations did not report broad memory deficits, except for reduced visual WM. In these cases, factors such as medication use and lower psychosocial functioning, markers of symptom severity, may contribute to the observed impairments [167]. A recent review concluded that individuals with GAD exhibit impairments in multiple cognitive domains, particularly in WM. These patients often show deficits in short-term memory and attentional control, with performance deteriorating in anxiety-inducing situations [145].

The role of pharmacological treatment in cognitive performance among individuals with GAD remains debated. A study comparing pharmacologically treated and drug-naïve young patients with GAD found generalized impairments in immediate verbal memory and both SM and LTM non-verbal memory, with the greatest deficits in those receiving SSRIs, suggesting a possible medication effect [168], although disagreement does exist: some reports indicate negative impacts [169], objective studies often failed to confirm these effects [170,171]. Indeed, evidence on the cognitive effects of SSRIs is mixed following treatment, challenging the assumption that SSRIs negatively impact cognition [172]. In addition, other studies demonstrated cognitive improvements in verbal, episodic, and working memory domains [173,174].

Overall, findings on memory functioning in GAD are mixed. Working memory deficits are most consistently found in tasks requiring high levels of attentional control, while simple short-term memory tasks often remain intact. Conversely, some studies using visuospatial working memory tasks fail to replicate the deficits reported with verbal material, highlighting modality-specific inconsistencies. These discrepancies likely reflect differential activation of worry-related cognitive load, which disproportionately affects tasks requiring sustained inhibition rather than basic retention [155,160,168].

#### 3.3.2. Panic Disorder

The literature examining memory functioning in PD is heterogeneous and, at times, conflicting. Poorer performance on memory tasks has been consistently associated with PD [175], together with cognitive impairments including deficits in visual memory, spatial WM, STM, and LTM, and both verbal and non-verbal domains [176,177,178,179,180]. However, a systematic review found no consistent differences between individuals with PD and healthy controls across different memory domains, including verbal and visual memory, both short- and long-term, as well as WM [181,182,183]. Psychophysiological features such as elevated arousal and hyperventilation, reflected in increased skin conductance, frequent spontaneous fluctuations, and slow-wave EEG activity, may impair selective attention and contribute to these deficits [179]. Neurophysiological studies also showed altered P300 amplitudes, suggesting dysfunction within limbic–reticular circuits [184]. Moreover, individuals with higher panic symptom severity exhibit reduced LPP amplitudes to negative stimuli and diminished modulation of LPP by WM load, indicating attenuated processing of negative information alongside excessive reactivity to neutral stimuli, patterns consistent with impaired top-down attentional control [185]. Notably, these impairments were observed irrespective of chronic benzodiazepines (BZDs) use, supporting the hypothesis that cognitive deficits may be intrinsic to PD rather than solely attributable to pharmacological side effects [178]. Nevertheless, the potential contribution of BZDs to cognitive deficits remains debated, as several studies documented a link between BZD use and widespread memory impairments [186,187], and others found no significant associations [188,189]. This inconsistency limits definitive conclusions regarding the cognitive risks associated with BZDs use, highlighting the need for more controlled and longitudinal research to disentangle the relative contributions of pharmacological treatment and disorder-specific neurocognitive alterations.

Further evidence suggests that individuals with agoraphobia comorbid with PD exhibit significant EM deficits, affecting both free recall and cued recall [164]. These difficulties appear to reflect encoding deficits. Moreover, agoraphobia alone has been independently associated with increased risk of cognitive decline and poorer memory performance [175] and deficits in VSWM, particularly when tasks require allocentric spatial judgments [190].

Findings in panic disorder remain highly inconsistent across memory domains. Although several studies report deficits in episodic recall and visuospatial working memory, others using parallel testing formats fail to reproduce these deficits. Performance often varies depending on physiological arousal during testing, suggesting that state-related hyperarousal may amplify deficits in some paradigms but not others. This task- and state-dependence contributes to the heterogeneous cognitive profile observed in PD [164,177,180].

#### 3.3.3. Social Anxiety Disorder

Several studies reported no significant differences in verbal or non-verbal memory performance between individuals with SAD and healthy control groups [191,192]. By contrast, some evidence suggests that individuals with SAD may exhibit better cognitive performance in specific memory domains. Among these, enhanced VSWM capacity was reported [154], along with a tendency to retrieve autobiographical memories with greater specificity, particularly those associated with negative emotional content [193]. Specifically, research indicated that patients with GAD and SAD exhibit impairments related to both the duration and load of WM. It has been proposed that distinct anxiety disorders may be associated with specific domains of WM dysfunction, suggesting them as potential screening markers [194]. In addition, WM would represent a moderator in the relationship between anxiety and cognitive task performance, possibly explaining inter-study variability [161,195]. These findings suggest that socially aversive memories may be more readily accessible and perceived as more distressing and intrusive than in controls [193].

Conversely, other studies documented memory impairments in individuals with SAD. Deficits were reported in short-term verbal and visual memory tasks [176,196], as well as in EM, with significant impairments emerging in both free and cued recall (F (1202) = 7.30, *p* = 0.007) [164]. In a WM task involving both neutral and threat-related words, individuals with SAD performed more poorly on neutral stimuli, suggesting a general reduction in both visual and verbal WM capacity under sequential presentation [197]. However, these findings were not replicated in a subsequent study using the same method, where high social anxiety was instead associated with reduced WM performance specifically for threat-related stimuli [198].

Higher levels of social anxiety have been uniquely associated with enhanced LPP responses to negative stimuli during WM tasks, which are considered a neural marker of attentional control [185].

Neuroimaging evidence indicates reduced activation in the DLPFC as well as reduced deactivation in the ventrolateral prefrontal cortex (VLPFC) and PCC in relation to WM load [155].

Overall, evidence on memory functioning in SAD remains inconsistent. While some studies show greater specificity for negative autobiographical memories and preserved visuospatial working memory, others report reduced performance in both verbal and nonverbal short-term tasks. Furthermore, the impairments observed in sequential WM paradigms are not consistently replicated in block tasks, highlighting the influence of stimulus timing and emotional salience. These discrepancies suggest that cognitive performance in SAD is highly context-dependent and modulated by subject-relevant threat cues present in specific task designs [164,194,196].

#### 3.3.4. Other Anxiety Disorders

To the best of our knowledge, empirical research on memory functioning in other anxiety disorders remains limited, with much of the available evidence focused on pediatric populations. In youth with anxiety disorders such as separation anxiety disorder (SeAD), studies reported impairments in verbal and semantic memory and VSWM, perhaps related to reduced attentional flexibility and rigid cognitive schemas [158,199]. Children with SeAD show impairments in visual memory, which may stem from either dysfunctional memory and anxiety brain circuits (e.g., medial temporal lobe) or attentional biases during the encoding process [196], consistent with the “Attentional Control Theory” [125]. Findings from a sample of previously institutionalized youths would suggest that WM may function as a protective factor against the development of SeAD [200]. No significant EM impairments have been observed in young individuals with specific phobias [164].

Studies on separation anxiety disorder consistently document deficits in visuospatial and semantic memory in children, but comparable paradigms in adolescents and adults often fail to demonstrate similar deficits. Furthermore, while visual recall tasks often reveal weaknesses, recognition-based formats produce mixed or no results. In specific phobias, memory performance is largely preserved, although some studies find mild deficits in tasks involving phobia-related stimuli, suggesting a stimulus-specific rather than generalized deficit [158,199].

### 3.4. Memory in OCD

Obsessive–compulsive disorder is a psychiatric condition characterized by intrusive thoughts, images, or impulses (obsessions) and repetitive behaviors (compulsions), enacted to reduce distress or prevent feared outcomes [109].

The relationship between STM and OCD remains debated. While some early studies indicated mild STM deficits, most recent evidence suggests no substantial differences compared to healthy controls [201], so that both verbal and visuospatial STM appear relatively preserved [202,203,204]. However, executive dysfunctions frequently reported in OCD (e.g., planning and cognitive flexibility impairments) may limit the effective use of stored information (4). Therefore, memory in OCD is often considered structurally intact but functionally compromised, primarily due to attentional biases and inefficient encoding strategies [202,203,205,206]. By contrast, WM deficit has been more consistently associated with OCD. While early studies suggested minimal impairments [207], more recent and methodologically robust investigations have identified significant dysfunctions, particularly in VSWM [208,209,210,211]. Notably, these impairments appear more pronounced among female patients, possibly reflecting neurobiological or phenotypic differences, such as the higher prevalence of symmetry-related compulsions in women, which may impose greater demands on VSWM. They are often attributed to dysfunctions in executive control processes, including the ability to update, manipulate, and suppress information [212]

Some researchers argue that WM dysfunction in OCD is not primarily due to difficulties in inhibiting intrusive thoughts, but rather to impairments in discarding no-longer-relevant information, leading to cognitive overload [211,213]. Neuroimaging studies reported abnormal activation of the DLPFC during WM tasks [214], while genetic studies related OFC activity with genetic susceptibility to OCD during memory processing [215]. These findings support the view that WM deficits in OCD are rooted in executive dysfunctions, which may also underlie compulsive behaviors [216].

Episodic memory deficits represent one of the most consistently documented cognitive abnormalities in OCD, particularly affecting both encoding and retrieval phases during tasks requiring delayed recall and complex organizational strategies [164,205,212]. They might stem from inefficient encoding strategies and heightened metacognitive monitoring (i.e., excessive scrutiny of one’s cognitive processes), which may interfere with consolidation [217]. In directed forgetting paradigms, patients show reduced capacity to inhibit irrelevant information, suggesting an encoding-related overload. Gender differences have also been reported, with male patients exhibiting greater impairments in episodic and organizational memory than females [218].

Functional neuroimaging studies revealed hypoactivation of the DMPFC and hyperactivation of the PCC, regions involved in self-referential processing and memory retrieval that might contribute to the inefficient modulation of episodic memory networks [219].

Prospective memory is also impaired in OCD, particularly in subclinical populations, with greater deficits observed during tasks involving neutral stimuli [220,221,222,223,224]. However, time-based PM relies more heavily on self-initiated retrieval and sustained attention, rendering it particularly vulnerable to individual differences in executive functioning [224]. Neurofunctional studies corroborate this, showing reduced DLPFC activation during both anticipation and execution phases of PM tasks [225].

By contrast, retrospective memory appears generally preserved in individuals with OCD [226]. However, those with compulsive checking behaviors, a distinct phenomenon known as “memory distrust” emerges, wherein individuals express diminished confidence in their memory and reduced vividness of recollection despite intact objective performance [227,228,229]. This phenomenon may operate bidirectionally: compulsive checking undermines memory confidence, which in turn reinforces the compulsion [230,231].

In this disorder, there is a clear dissociation between structurally intact short-term and retrospective memory and deficits that emerge in tasks requiring strategic encoding, delayed retrieval, or manipulation of complex information. In particular, visuospatial working memory deficits appear robust in some paradigms, but similar deficits are not consistently reproduced in computerized n-back tasks, suggesting a task-specific sensitivity. Similarly, episodic memory performance varies across studies depending on the degree of organizational strategy required, strengthening the interpretation that executive and metacognitive factors determine the observed inconsistencies [203,204,211].

### 3.5. Memory in PTSD

Post-traumatic stress disorder is a psychiatric condition that develops in individuals who have either directly or indirectly experienced or witnessed one or more traumatic events (such as threats to life, severe injury, or violence [232,233]. Its core symptoms include intrusions (e.g., intrusive memories, nightmares, flashbacks), avoidance of trauma-related stimuli, negative alterations in cognition and mood (e.g., guilt, emotional numbing), and hyperarousal (e.g., irritability, insomnia, hypervigilance) [234]. Beyond affective and behavioral manifestations, PTSD is increasingly conceptualized as a disorder involving widespread memory dysfunction. These deficits extend beyond the recall of specific traumas to affect working memory, long-term episodic memory, autobiographical narration, and the ability to plan for the future [235,236].

Deficits in working memory are among the most consistently reported [235,236,237]. Patients show reduced accuracy in verbal and VSWM tasks, reflecting difficulties in maintaining and manipulating short-term information [236]. These impairments correlate with symptoms of intrusion and hyperarousal, suggesting that weakened executive control contributes to an inability to suppress traumatic thoughts [238,239,240]. In veterans, VSWM deficits accounted for ~30% of the variance in re-experiencing symptoms, highlighting the role of spatial processing in flashbacks [241]. Working memory dysfunction is linked to reduced DLPFC efficiency, limiting adaptive regulation of traumatic memories [242,243]. Importantly, these deficits are not only due to emotional hyperarousal or comorbidity with MDs, but are also associated with slower information processing [244,245], while limiting the brain’s capacity to update WM and exacerbating emotional regulation and amplifying susceptibility to intrusive memories [246].

Episodic memory is significantly impaired, particularly for verbal tasks [235,236,247]. A meta-analysis confirmed that deficits extend beyond trauma-related content to encompass neutral everyday events [235]. Event segmentation studies show that patients encode daily experiences less coherently, segment actions less effectively, and recall fewer details, with disorganized event coding accounting for nearly half of EM deficits [248]. However, findings vary depending on trauma type, comorbidities, and assessment tools [249,250].

Autobiographical memory, crucial for identity and coherence of life narratives [251,252], is also compromised. Traumatic memories often appear fragmented, disorganized, and sensory-laden, resisting integration into coherent narratives [253,254]. Debate persists over whether these alterations are direct consequences of trauma-related brain changes or reflect pre-existing vulnerabilities. [255,256].

From a neurobiological perspective, PTSD has been associated with reduced hippocampal volume, hyperactivation of the amygdala, and underactivity of the prefrontal cortex [257]. These factors contribute to deficits in memory consolidation and retrieval processes [255,256,258]. According to the “Dual Representation Theory”, traumatic memories are encoded mainly in sensory, lacking coherent narrative context, which results in fragmented, intrusive recollections [259]. Neuroimaging supports these findings, and disrupted amygdala-prefrontal connectivity impairs emotional regulation [260].

Disrupted sleep in PTSD hinders normal memory consolidation, while preserving emotional intensity [261,262]. Reconsolidation offers intervention opportunities, as propranolol, cognitive tasks, and “Targeted Memory Reactivation” show promise in reducing flashbacks [263,264].

In this context, narrative-based interventions have proven particularly effective, as they might also trigger beneficial epigenetic effects [265,266].

In conclusion, PTSD is marked by profound disruptions in episodic and autobiographical memory, against a backdrop of divergent findings across recognition paradigms, with several studies reporting preserved performance despite marked deficits in free recall. This pattern reflects varying sensitivity to impaired event segmentation and contextual binding. Furthermore, working memory performance varies substantially across tasks; visuospatial working memory deficits are robust, while verbal working memory performance is inconsistent, likely due to differences in emotional load across paradigms. These discrepancies highlight the need for more standardized working memory measures in PTSD research [235,236,242,253].

### 3.6. Memory in Psychotic Disorders

Psychotic disorders represent a heterogeneous group of severe psychiatric conditions characterized by profound disturbances in perceptual, cognitive, and affective domains, culminating in a marked disconnection from shared reality [109]. These syndromes are defined by hallmark symptoms including delusions, hallucinations, disorganized speech, grossly disorganized or catatonic behavior, and, in more severe or chronic cases, negative symptoms such as apathy, alogia, and anhedonia [109]. Emerging evidence suggests that alterations in memory function may be intrinsically linked to the disruption of reality processing observed in psychotic disorders. One prevailing hypothesis posits that memory deficits, particularly AM and EM, may contribute to or exacerbate the impaired reality monitoring and self-experience typical of psychosis [267,268]. Several studies consistently demonstrated that both individuals diagnosed with schizophrenia and those presenting subclinical psychotic traits exhibit significant difficulties in constructing and maintaining a temporally coherent and contextually anchored sense of self. However, the precise cognitive and neurobiological mechanisms underlying this disturbance in self-representation remain incompletely elucidated [37,42].

#### 3.6.1. Schizophrenia

Schizophrenia is characterized by pervasive cognitive impairments, with memory deficits and functioning representing a core feature of its neuropsychological profile [269]. Working memory is particularly affected [270]. Patients consistently show deficits in verbal and visuospatial maintenance tasks [271], and a recent narrative review highlighted reduced accuracy and slowed responses in WM tasks [269]. Importantly, such deficits may precede the onset of the disease by several years [272]. Functional neuroimaging studies show reduced BOLD activity in the superior parietal lobule, DLPFC, and hippocampus [271,273], with DLPFC activation following an “inverted U-shaped” pattern [269]. Collectively, these findings suggest hypofunction of the frontal and parietal cortex during memory tasks [271,273], reflecting inefficient neural recruitment [269]. At neurobiological level, abnormalities in cortical D1 transmission in the DLPFC, and imbalances in the GABA-glutamate system have been proposed, potentially compromising the stability of prefrontal signaling during information retention [274,275]. Short-term memory is also impaired. A meta-analysis found that patients perform significantly worse on verbal and spatial tasks compared to healthy controls, indicating a reduced capacity to passively maintain information without active manipulation [270]. Long-term memory deficits are likewise prominent, with EM particularly compromised in free recall and, to a lesser extent, recognition [276], possibly reflecting failures in strategic encoding and abnormal retrieval rather than consolidation problems [277]. Neuroimaging studies show reduced activation of the DLPFC and hippocampus during recall [278,279], while incidental encoding remains relatively intact [277]. Structural neuroimaging analyses further reveal reduced hippocampal volumes, particularly in anterior regions [280]. Prospective and retrospective memory is also impaired, especially in time- and event-based tasks, where patients make more errors in stimulus monitoring, possibly related to prefrontal-striatal circuits [279,281]. Semantic memory is also affected, although typically less severely than EM [276,282]. Patients show reduced semantic fluency reflecting both executive difficulties and impairments in lexical retrieval [283,284,285,286]. These deficits likely involve temporal and frontal cortical dysfunction, although research remains limited. By contrast, procedural memory appears relatively preserved: automatic perceptual–motor skills mediated by the basal ganglia and motor cortex remain largely intact, although more complex forms of conceptual or semantic-based procedural learning may be subtly impaired [287,288]. Importantly, memory impairments in schizophrenia are largely independent from positive symptoms, albeit correlating with negative and disorganization symptoms [276,289]. This may explain why traditional antipsychotics, although effective against positive symptoms, have little impact on memory recovery [289,290]. Moreover, anticholinergic medications used for negative symptoms can further worsen memory due to their cognitive side effects [291,292]. Attempts with pro-cognitive drugs such as donepezil or memantine have yielded disappointing results [289,290]. In contrast, psychotherapeutic approaches, including cognitive remediation, targeted cognitive training, and memory-focused therapies, have shown modest but promising benefits [290].

Working memory deficits are consistently replicated across modalities and paradigms, yet short-term memory results are less uniform. While backward digit span is consistently impaired, forward digit span shows more heterogeneous results. Episodic memory deficits also vary depending on task structure, with free recall consistently impaired but recognition less so, indicating a disproportionate vulnerability of strategic retrieval processes. Furthermore, procedural learning is preserved in tasks involving basic motor sequencing, yet more complex probabilistic learning paradigms have produced conflicting results, suggesting that “procedural preservation” may be domain-specific rather than universal [275].

#### 3.6.2. Other Psychotic Disorders

Memory deficits extend across the psychosis spectrum, though with varying severity. Overall cognitive profiles are broadly similar, but distinct features emerge between disorders [293]. Deficits in attention, processing speed, memory, and WM are quite severe in schizophrenia, intermediate in psychotic BD and least pronounced in psychotic depression [294]. Verbal memory impairment appears to be a common denominator, whereas processing speed deficits are more typical of schizophrenia, and executive impairments are more pronounced in bipolar psychosis [294]. In delusional disorder, memory deterioration is more global, affecting all phases of memory [295]. Memory impairments are already detectable in the prodromal phase [275] and are present at the first psychotic episode at levels comparable to those of chronic schizophrenia, with further decline occurring in advanced age or severe illness [275,296]. Moreover, even asymptomatic relatives show subtle memory deficits, suggesting that genetic or familial vulnerability factors contribute to these impairments [275]. Altogether, these findings support memory dysfunction as a transdiagnostic marker of psychosis, with neuroimaging implicating disruptions in prefrontal–limbic network and dopaminergic/glutamatergic balance [297,298].

In summary, memory dysfunction represents a core and pervasive feature of schizophrenia, with marked impairments in working, episodic, prospective, and semantic memory, while procedural memory remains relatively preserved. These deficits are strongly linked to prefrontal–hippocampal dysfunction and correlate with negative and disorganization symptoms and show limited responsiveness to pharmacological interventions but partial improvement with cognitive remediation. Across the psychosis spectrum, memory impairments are detectable already in prodromal stages, vary in severity between disorders, and likely reflect shared vulnerability factors, consolidating their role as a transdiagnostic marker of psychosis.

Although verbal memory deficits are widely shared across psychotic conditions, the extent and pattern of deficits differ considerably. Psychotic depression often shows milder deficits, while delusional disorder presents more comprehensive deficits in encoding, storage, and retrieval. In bipolar psychosis, high-executive working memory tasks are most affected, while simpler tasks often remain intact, producing a mixed pattern not observed in schizophrenia. These comparative inconsistencies underscore the need for harmonized cognitive batteries across diagnoses across the psychotic spectrum [295,297].

### 3.7. Therapeutic Strategies Addressing Memory Deficits in Psychiatric Disorders

As previously mentioned, memory deficit could represent a core transdiagnostic feature across different neuropsychiatric disorders, particularly MDD, BDs and schizophrenia. These deficits are associated with significant impairments in daily functioning, quality of life, and social functioning [139,299]. Consequently, the recovery of cognitive functions could be recognized as a strategic objective within integrated rehabilitation programs designed to promote autonomy, counteract chronicity, and support sustainable recovery [300,301].

A multidimensional approach that integrates pharmacotherapy, psychotherapy, cognitive training, and psychosocial interventions may currently be considered the most effective strategy for the integrated treatment of these conditions [301]. Within this framework, therapeutic choices can be more explicitly linked to the characteristic profiles of memory impairment observed in each disorder, thereby aligning interventions with underlying neurocognitive dysfunctions.

From a pharmacological perspective, selective serotonin reuptake inhibitors (SSRIs) have been studied for their potential neurogenic effects, which may enhance brain plasticity and improve specific cognitive functions [171]. In major depressive disorder, where episodic memory and processing speed are often compromised, these agents may exert indirect benefits by normalizing fronto-limbic functioning associated with affective interference on memory encoding. Other agents, such as second-generation antipsychotics, glutamatergic modulators (e.g., glycine, D-serine, D-alanine), cholinesterase inhibitors (donepezil, galantamine), and sympathomimetics (e.g., modafinil), have shown promising results in improving cognitive performance, though the evidence remains partial and sometimes conflicting [302]. In schizophrenia, these agents may specifically target fronto-hippocampal inefficiency and NMDA-receptor–related deficits that underlie impairments in working memory and learning.

Psychotherapeutic interventions also play a complementary role in cognitive recovery. Cognitive-Behavioral Therapy (CBT) has been shown to positively influence mnemonic processes by strengthening functional connectivity between frontal and limbic brain regions, enhancing the integration of cognitive and affective domains [303,304]. In disorders such as MDD and BD, where negative self-schemas and mood-congruent recall distort autobiographical memory, CBT may enhance memory retrieval by promoting more coherent and regulated cognitive-emotional processing.

Within this framework, increasing attention is focused on innovative and complementary strategies to enhance memory and cognitive functions. Among these, non-invasive brain stimulation techniques, such as transcranial direct current stimulation (tDCS) and repetitive transcranial magnetic stimulation (rTMS), have shown some promise in modulating synaptic plasticity and activating the fronto-temporal networks involved in working memory and mnemonic encoding [301,305]. Simultaneously, immersive virtual reality (VR) tools are being used to train EM and PM in ecologically valid settings, simulating real-life situations and facilitating the transfer of cognitive skills to daily [306,307]. This is especially pertinent in disorders where prospective memory failures, such as forgetting planned actions, represent an obstacle to functional autonomy.

In keeping with the pharmacological subsection above, we briefly consider those compounds that may be deemed innovative on the grounds of a novel mechanism of action and the availability of human evidence on memory—or, more broadly, on cognition—in major psychiatric disorders; preclinical-only signals were therefore not taken into account. Within antidepressant strategies, the multimodal serotonergic agent vortioxetine has repeatedly shown advantages on processing speed and learning/working memory in major depression, with effects that appear only partly mediated by mood improvement [308,309]. These findings resonate with the attentional and executive components of memory impairment that often characterize MDD. Turning to glutamatergic modulation, sodium benzoate—acting as a D-amino-acid oxidase inhibitor and thereby enhancing NMDA co-agonism—has yielded add-on improvements on composite neurocognitive batteries in schizophrenia, alongside symptomatic gains [310]. In the glycinergic domain, inhibition of the glycine transporter-1 with iclepertin (BI-425809) produced Phase-2 benefits on the MCCB cognitive composite in schizophrenia, while larger Phase-3 programs have provided mixed results and call for a cautious interpretation [311]. Cholinergic approaches illustrate the volatility typical of this field: the α7-nicotinic partial agonist encenicline (EVP-6124) showed early cognitive and functional signals that were not subsequently replicated at Phase-3, thereby tempering initial expectations [312,313]. Finally, pleiotropic neurotrophic strategies such as recombinant human erythropoietin have demonstrated benefits on memory and processing speed in small randomized trials across schizophrenia and mood-disorder samples, although their use presupposes appropriate hematological monitoring and awaits broader confirmatory evidence [314,315]. These pharmacological avenues converge on mechanisms, glutamatergic, serotonergic, cholinergic, and neurotrophic, that are central to the neural circuits supporting episodic and working memory across psychiatric populations.

In conclusion, memory impairment, when observed in severe psychiatric disorders, may represent a critical clinical challenge with profound implications for daily functioning and personal autonomy. Current evidence suggests that an integrated, multimodal approach combining pharmacological, psychotherapeutic, and cognitive rehabilitation interventions may constitute the most promising strategy to effectively address these deficits. By more closely mapping specific interventions onto the characteristic memory profiles of each disorder, such as fronto-hippocampal dysconnectivity in schizophrenia, affectively biased autobiographical memory in mood disorders, or prospective memory failures across conditions, treatment can be better tailored to the underlying cognitive architecture. While further research is needed to clarify specific mechanisms and optimize personalized intervention, integrating neuroscience, clinical psychology, and advanced technologies offers new, tangible prospects for cognitive and functional recovery in individuals with complex mental disorders. Future research should continue to refine how specific interventions can be matched to well-characterized memory profiles across disorders, thereby improving the precision and clinical impact of therapeutic strategies.

## 4. Discussion

The present review highlights that memory dysfunction is a pervasive yet heterogeneous feature across major psychiatric disorders. A first interpretative distinction concerns the degree of robustness of the available results. Established evidence includes persistent working memory deficits in schizophrenia and bipolar disorder, replicated across different paradigms and supported by neurobiological data, reduced episodic/autobiographical specificity in major depression and PTSD, and relative preservation of procedural memory across disorders. In contrast, findings in anxiety disorders, OCD, and some subtypes of bipolar disorder remain more heterogeneous and context-dependent, often reflecting small sample sizes, variable task parameters, or inadequate control for comorbidities. Rather than being confined to a single diagnostic category, alterations in specific memory domains are present in different disorders, albeit with distinct profiles and underlying mechanisms. In MDD, impairments are particularly evident in EM and AM, often manifesting as overgeneralized recollections that restrict access to specific positive experiences and interfere with emotional regulation [79,81,85]. Such phenomena have been interpreted as both avoidance strategies and markers of hippocampal dysfunction in pattern separation, consistent with evidence of structural and functional anomalies in hippocampal-prefrontal circuits and altered connectivity within the default mode network [80,89,316]. Working memory dysfunction, another well-documented feature of MDD, appears to be driven by impaired executive control and attentional bias toward negative stimuli, while short-term memory deficits, though less pronounced, further contribute to daily functional difficulties [92,95,317]. Importantly, these deficits often persist beyond acute episodes, suggesting that memory dysfunction constitutes a core dimension of the disorder [78,82,84].

In BDs, cognitive impairment also emerges as a stable and clinically relevant feature. Deficits in verbal and visuospatial EM, WM, and autobiographical recall are consistently observed not only during acute phases but also in euthymic states, indicating a trait-like vulnerability that extends to unaffected first-degree relatives [116,120,131,133,318]. Neuroimaging findings implicate fronto-limbic dysfunction, with altered prefrontal and amygdala activation even during tasks unrelated to current mood state, and emotional load strongly modulates memory performance [123,131,133]. This evidence suggests that in this case, memory disturbances reflect the interaction between enduring neurocognitive vulnerabilities and state-dependent emotional dysregulation [123,133,142]

In anxiety disorders, memory outcomes are less consistent, but converging evidence supports the notion that WM is particularly sensitive to the effects of heightened arousal and worry. The “Attentional Control Theory” [125] provides a useful framework, positing that anxiety disrupts the balance between goal-directed and stimulus-driven attentional systems, thereby compromising the efficiency of working memory. Experimental studies show reduced accuracy and efficiency in both verbal and visuospatial tasks, especially under high cognitive load or threat-related conditions [155,319]. However, some findings report preserved or even enhanced performance in specific domains, such as visuospatial WM in SAD, indicating that anxiety-related cognitive alterations are highly context-dependent and influenced by symptom severity, task demands, and compensatory strategies [193,320]. Similarly, in PD and GAD, deficits in EM and WM have been described, but results remain controversial, partly due to methodological heterogeneity and the potential confounding role of pharmacological treatments, particularly benzodiazepines and SSRIs [168,178,321]. Overall, findings in anxiety suggest that memory deficits are heterogeneous and related to cognitive load, emotional salience, and regulatory control.

Obsessive–compulsive disorder presents a somewhat distinctive profile. While STM and RM are generally preserved, deficits in MW and EM are consistently reported, with impairments attributed to inefficient encoding strategies, heightened metacognitive monitoring, and executive dysfunction [205,206,212]. Importantly, the phenomenon of “memory distrust” in compulsive checkers illustrates how subjective confidence in memory can be decoupled from objective performance, reinforcing compulsive behaviors despite intact structural memory systems [228,229,322]. This suggests that memory dysfunction in OCD may be more functional than structural in nature, rooted in altered metacognitive processes rather than primary mnemonic deficits [230].

In PTSD, memory dysfunction is broad and profound, extending beyond trauma-related material to affect WM, episodic recollection, and autobiographical coherence. Traumatic memories are often fragmented and sensory-bound, consistent with dual representation theory, which distinguishes between sensory-based representations and verbally accessible memories [259,323]. Neurobiological findings converge on reduced hippocampal volume, amygdala hyperactivity, and prefrontal underactivation, leading to impaired contextualization and regulation of traumatic memories [243,260,324]. Moreover, disrupted sleep has been shown to interfere with systems-level consolidation, preserving the emotional salience of traumatic events and increasing susceptibility to flashbacks [261]. Event segmentation studies further indicate that patients encode daily experiences less coherently, contributing to disorganized recall and functional impairment [248]. These findings underscore the centrality of memory processes in the phenomenology of PTSD, where dysfunction in encoding, consolidation, and retrieval sustains core symptoms such as intrusions and avoidance [235,236].

Psychotic disorders, and, in particular, schizophrenia, exhibit the most pervasive and severe profile of memory impairments, with deficits in WM, EM, PM and SM consistently documented, while procedural memory remains relatively preserved [269,271,276,287]. Neuroimaging and neurochemical studies indicate that these impairments are closely linked to prefrontal–hippocampal dysconnectivity, reduced D1 receptor transmission in the dorsolateral prefrontal cortex, and imbalances in glutamatergic and GABAergic systems [275]. Importantly, memory deficits often precede the onset of psychosis and persist throughout the course of illness, supporting their role as potential endophenotypic markers. Moreover, they show limited responsiveness to antipsychotic treatment, which primarily targets positive symptoms, while cognitive remediation and targeted psychosocial interventions have shown modest but clinically meaningful benefits [289,290]. These findings reinforce the notion that memory dysfunction is not secondary but intrinsic to the pathophysiology of schizophrenia and related psychotic disorders. Across the psychosis spectrum, severity gradients are evident, most pronounced in schizophrenia, intermediate in psychotic BD, and milder in psychotic depression, with verbal memory deficits as a common denominator and disorder-specific variations in processing speed and executive function [293,294,325].

Taken together, the evidence supports a transdiagnostic view of memory dysfunction in psychiatry (Table 1, Table 2, Table 3, Table 4, Table 5 and Table 6). Across disorders, impairments cluster around WM control, episodic binding, and autobiographical specificity, but the mechanisms differ: affective bias and DMN hypercoupling in depression, fronto-limbic reactivity in BD and anxiety, trauma-driven consolidation failures in PTSD, and fundamental prefrontal–hippocampal inefficiency in schizophrenia [89,123,131,155,259,261,271,273,274,316]. Such commonalities and divergences underscore the importance of moving beyond categorical diagnoses toward a dimensional framework, focusing on cognitive control, salience processing, and mnemonic integration as core domains of dysfunction. From a clinical perspective, this suggests that interventions aimed at enhancing executive control, improving sleep-dependent consolidation, and restructuring dysfunctional metacognitive beliefs may have broad applicability across disorders. Promising avenues include cognitive remediation programs, non-invasive brain stimulation targeting prefrontal networks, virtual reality–based training for episodic and prospective memory, and reconsolidation-based approaches for trauma-related memories [290,301].

Memory domains and neural circuits included in Table 1 were identified based on convergent findings across studies using validated cognitive measures and consistent neuroimaging or neurobiological evidence.

The interpretability of existing findings is limited by several methodological weaknesses in the relevant literature. Many studies rely on small or clinically heterogeneous samples, often mixing acute and remission phases or failing to control for comorbid anxiety, substance use, or medication effects. Memory tasks vary greatly in their structure, emotional burden, and scoring methods, limiting the ability to draw disorder-specific conclusions. Furthermore, longitudinal data remain scarce, making it difficult to distinguish between state-dependent alterations and trait-like vulnerabilities. The lack of harmonized protocols also reduces replicability and increases variability between studies. Lastly, across disorders, variability in task parameters, illness phase, comorbidities, and medication use represent major sources of heterogeneity and likely contribute to the inconsistencies observed in the literature.

At the same time, the heterogeneity and inconsistency of current findings highlight the need for methodologically rigorous studies with larger samples, harmonized task batteries, longitudinal designs, and careful control of confounders such as medication and comorbidity. Ultimately, clarifying the mechanisms and moderators of memory dysfunction holds not only theoretical significance but also practical relevance for improving daily functioning, autonomy, and quality of life in individuals with psychiatric disorders. Furthermore, although a dimensional perspective is valuable for identifying shared mechanisms across disorders, it also has limitations. Dimensional models may obscure relevant disorder-specific features, and operational definitions often vary across studies, reducing comparability. In addition, some trajectories of illness, treatment response, and functional outcome remain more closely aligned with categorical distinctions. For these reasons, dimensional and categorical approaches should be viewed as complementary rather than mutually exclusive when investigating memory dysfunction in psychiatric disorders.

The novelty of this synthesis, despite the limitations noted above, lies in the integration of memory findings from different psychiatric categories and from declarative, nondeclarative, and prospective systems, thus highlighting transdiagnostic patterns not evident in disorder-specific reviews. By mapping shared neural substrates, particularly prefrontal-hippocampal disconnection, and linking them to cognitive and clinical manifestations, the review offers a dimensional perspective that complements traditional categorical approaches.

## 5. Conclusions

Across the psychiatric conditions reviewed, memory dysfunction appears as a prominent, though not primary, transdiagnostic dimension with variable expression across disorders. While it does not occupy the defining role observed in neurocognitive disorders, converging evidence indicates that specific memory alterations may contribute to clinical burden and functional outcomes, in interaction with other cognitive and affective processes. While impairments cluster around EM and WM, the expression of these deficits varies, reflecting the interplay between structural alterations, network dysregulation, emotional modulation, and metacognitive processes. These findings suggest that memory dysfunction is not merely a secondary consequence of psychiatric illness, but rather a core dimension that contributes to symptom persistence, functional disability, and reduced quality of life. Clinically, this underscores the importance of incorporating cognitive assessment and targeted remediation into standard psychiatric care, alongside pharmacological and psychotherapeutic approaches. As no formal quality assessment or structured screening procedure was applied, the synthesis may be subject to selection bias and relies on the convergence of findings across heterogeneous methodologies and clinical samples. Our review offers a unique perspective by framing memory dysfunction as a transdiagnostic dimension, with convergent patterns and shared neurobiological mechanisms across major psychiatric disorders. The qualitative nature of the review also limits the ability to draw firm causal inferences or quantify the relative magnitude of memory alterations across disorders. These constraints highlight the need for more systematic and methodologically harmonized research. Future studies should prioritize: (i) longitudinal and multimodal designs to clarify when and how specific memory domains become altered across illness trajectories; (ii) harmonized cognitive batteries and standardized diagnostic criteria to reduce heterogeneity and clarify the role of emotional load in working memory across anxiety disorders; (iii) mechanistic investigations linking neural circuitry, memory processes, and symptom expression across disorders; and (iv) the development of targeted interventions, both cognitive and neurobiological, that specifically address identified memory vulnerabilities. Addressing these priorities may advance a more integrated understanding of memory dysfunction and inform personalized treatment strategies.

## Figures and Tables

**Table 1:** Summary of key memory profiles (affected domains and main neural circuits) across all reviewed disorders.

Disorders	Affected Domains	Neural Circuits
Major Depressive Disorder	EM, WM, and AM	Hippocampal dysfunction, structural and functional anomalies in hippocampal-PFC circuits, altered connectivity in default mode network
Bipolar Disorders	EM, WM, and AM	Fronto-limbic dysfunction, altered attivation in PFC and amygdala
Anxiety Disorders	EM and WM	Deactivation and decrease white matter in PFC, amygdala and PFC increase connectivity, altered activation of PFC, PPC, and ACC particularly in dx hemisphere
Obsessive–compulsive Disorder	WM and EM	PFC and OFC deactivation, PCC iperactivation
Post-Traumatic Stress Disorder	WM, EM and AM	Reduced hippocampal volume, amygdala hyperactivity and PFC underactivation
Psychotic Disorders	WM, EM, PM and SM	Hippocampal-PFC dysconnectivity, reduced D1 receptor transmission in DLPFC, imbalances in glutamatergic and GABAergic systems

***Legend*** *. ACC, anterior cingulate cortex; AM, autobiographic memory; DLPFC, dorsolateral prefrontal cortex; EM, episodic memory; OFC, orbitofrontal cortex; PCC, posterior cingulate cortex; PFC, prefrontal cortex; PM, prospective memory; PPC, parietal posterior cortex; SM, semantic memory; WM, working memory.*

**Table 2:** Summary of relevant studies examining cognitive and neural correlates of memory and executive functioning in Major Depressive Disorder (MDD) and Bipolar Disorder (BD).

*MDD*			
*Authors*	*Type of Study*	*Tasks*	*Results*
Bremner et al., 2004 [316]	fMRI N = 27 (MDD = 18; HC = 9)	Verbal Memory Encoding Task	↓ Hippocampal and DLPFC activation during encoding and retrieval (*t* = 6.52, *p* < 0.0001)
Dietsche et al., 2014 [80]	fMRI N = 46 (MDD = 23; HC = 23)	Encoding and Recognition task Verbal Learning and Memory Test (VLMT)	Fronto-limbic disconnection; ↓ activity in DLPFC and ACC (Encoding: *t* = 1.77, *p* < 0.08; Recognition: *t* = 2.68 *p* < 0.01)
Jayaweera et al., 2016 [83]	Case–control N = 111(MDD = 84; HC: 27)	Verbal episodic memory Rey Auditory Verbal Learning Test and Logical Memory (WMS-III)	Smaller right anterior caudate (*t* = 2.3, *p* = 0.026) and poorer verbal memory (*t* = 2.5, *p* < 0.001); smaller caudate associated with worse memory (r = 0.3, *p* = 0.003)
Butters et al., 2009 [172]	fMRI N = 38 (MDD = 23; HC: 15)	Caudat volumetry and verbal learning	Smaller caudate correlates with poor verbal recall in geriatric MDD (*t* = 2.23; *p* 0.032; r = 0.35)
Harvey et al., 2005 [99]	fMRI (N = 20; MDD = 10; HC = 10)	Verbal n-back task	LPFC and ACC hyperactivation
Walter et al., 2007 [100]	fMRI (N = 20; MDD = 12; HC = 17)	Verbal WM task	↑ Left DLPFC (Z = 3.90) and ↓ MPFC (Z = 4.48)
Fitzgerald et al., 2008 [87]	fMRI (N = 26; MDD = 13; HC = 13)	Tower of London task and n-back task	↑ Right prefrontal activation across cognitive tasks (*t* = 2.5, *p* < 0.05)
Wachowska et al., 2022 [84]	Case–control (N = 87; MDD = 50; HC = 30)	Cytokine assays and episodic tasks	↑ IL-1β and IL-6; no clear link with memory deficits
***BD***			
Deckersbach et al., 2004 [132]	Case–control (N = 90; BD = 30; OCD = 30; HC = 30)	CVLT	Impaired encoding and retrieval in BD; partial compensation through verbal organization
Deckersbach et al., 2008 [131]	fMRI (N = 26; BD = 9; HC = 17)	2-back working memory paradigm	↑ left DLPFC (BA9/46) activation during sadness (*t* = 2.59, *p* = 0.02); ↑ dorsal ACC across conditions
Glahn et al., 2010 [142]	Case–control (N = 660; BD = 230; HC = 230)	Digit Symbol Coding Task, Object Delayed Response, and immediate facial memory.	Cognitive impairments genetically correlated with BD risk (ρg = −0.44 to −0.53, *p* = 0.03–0.009) and intercorrelated across tasks (ρg = 0.59–0.78, *p* ≤ 0.003).
King et al., 2013 [133]	Case–control (N = 20; BDI = 12; BDII = 8)	Autobiographical Memory Interview	Overgeneral and vague recall with mood-congruent bias; ↓ retrieval of positive events (*t* = 2.33–2.56, *p* < 0.05)
Mullin et al., 2012 [123]	fMRI (N = 41; BDI = 22; HC = 19)	Emotional n-back	↓ dlPFC, dACC, parietal and putamen activity in 2-back; ↑ left dlPFC and amygdala during fearful distracters (*p* < 0.05, corrected)
Bertocci et al., 2011 [122]	fMRI (N = 57; UDD = 23; BDI: 28; HC: 16)	Emotional face n-back	UDD > BD > HC in left dAMCC and putamen activation (*t* = 3.06–2.95, *p* < 0.05); group effects in putamen (F = 6.8–7.7, *p* < 0.05)
Liu et al., 2010 [127]	Comparative fMRI (N = 48; BDI: 14; BDII: 13 HC: 21)	WCST, WMS, TAP	BDI: ↓ FA in right subgenual ACC linked to poor recall (ρ = −0.83, *p* < 0.001); BDII: broader emotional-cognitive deficits (ρ = −0.39–0.47, *p* < 0.05)

Arrows indicate directionality of findings: ↑ = increased activation/levels; ↓ = decreased activation/levels. **Legend:** *ACC, anterior cingulate cortex; AMT, Autobiographical Memory Test; BD, Bipolar Disorder; BDI/BDII, Bipolar Disorder type I/type II; CVLT, California Verbal Learning Test; DLPFC, dorsolateral prefrontal cortex; dlPFC, dorsolateral prefrontal cortex (lowercase notation retained from original paper); dACC, dorsal anterior cingulate cortex; FA, fractional anisotropy; fMRI, functional magnetic resonance imaging; HC, healthy controls; IL-1β/IL-6, interleukin-1 beta/interleukin-6; LPFC, lateral prefrontal cortex; MDD, Major Depressive Disorder; MPFC, medial prefrontal cortex; OCD, obsessive–compulsive disorder; r, Pearson’s correlation coefficient; ρ, Spearman’s rho; t, Student’s t-test; F, F statistic; WM, working memory; WMS, Wechsler Memory Scale; WCST, Wisconsin Card Sorting Test; VLMT, Verbal Learning and Memory Test; UDD, unipolar depressive disorder; TAP, Test for Attention Performance.*

**Table 3:** Summary of relevant studies investigating memory performance in anxiety disorders.

*SAD*			
*Authors*	*Type of Study*	*Tasks*	*Results*
Abushalbaq et al., 2021 [194]	Case-control N = 82 (SAD = 20; PD = 18; GAD = 20; HC = 24)	Working memory tasks: NAART; Digit Span (WAIS-R); Short/long-delay; N-back	WM impairments related to both duration and load (d = 0.65–0.80, *p* < 0.05)
Vasa et al., 2006 [196]	Case–control N = 160 (SAD = 22; other anxiety disorder = 35; HC = 103)	Verbal/visual short-term recall task	Significant STM and episodic memory deficits. (F(4,87), *p* = 0.005)
Airaksinen et al., 2005 [164]	Case–control N = 287 (SAD = 32; PD = 33; GAD = 7; SP (specific phobia) = 24; OCD = 16; HC = 175)	Episodic memory task: free and cued recall	Significant impairments in episodic recall for SAD participants (Free recall: F(1,202) = 7.75, *p* = 0.006 Cued recall: F(1,202) = 7.30, *p* = 0.007)
***PD***			
Lucas et al., 1991 [180]	Case–control N = 50 (PD = 25; HC = 25)	Verbal and visual memory task	Impaired visual learning, visual recall, and verbal recall in PD patients for verbal and visual memory (F(13,36) = 2.66, *p* < 0.01)
Boldrini et al., 2005 [177]	Case–control N = 55 (PD/A = 15; OCD = 25; HC = 15)	Visual-spatial memory task: RCFT	Impairment in spatial learning (*p* = 0.028)
Airaksinen et al., 2005 [164]	Case–control N = 287 (SAD = 32; PD = 33; GAD = 7; SP (specific phobia) = 24; OCD = 16; HC = 175)	Episodic memory: free and cued recall	Significant impairments in episodic recall in PD patients Free recall: F(1,203) = 8.66, *p* = 0.004 Cued recall: F(1,203) =5.31, *p* = 0.022
***GAD***			
Vytal et al., 2013 [160]	Case-control N = 60 (GAD = 30; HC = 30)	Verbal WM task: N-back	Persistent WM impairments even under low load (F(2,118) = 58, *p* = 0.001, η^2^ = 0.21)
Balderston et al., 2017 [155]	Case-control N = 64 (GAD = 7; SAD = 3; GAD/SAD = 13; HC = 41)	WM task: N-back (safe and threat conditions)	Reduced accuracy and slower reaction times in both conditions (Reaction time: *t*(487) = 3.39; *p* < 0.01 Accuracy: *t*(487) = 3.36; *p* < 0.01)
Tempesta et al., 2013 [168]	Case-control N = 71 (GAD = 40; HC = 31)	Verbal and non-verbal memory tasks (drug-naïve vs. SSRI): Digit Span; CBTT; ROCF	Impairments in immediate verbal memory and SM/LTM non-verbal memory in SSRI users (F^a^ = 0.96 to 0.81)
***Other Anxiety Disorders***			
Toren et al., 2000 [199]	Case-control N = 33 (SeAD and/or OAD = 19; HC = 14)	Verbal and semantic memory task: California Verbal Learning Test (CVLT)	Impairments in verbal and semantic memory (F(10,20) = 3.26, *p* < 0.05)
Sbicigo et al., 2020 [158]	Cross-sectional N = 54 (SeAD = 45; GAD = 41; SAD = 17)	Visuospatial WM and episodic memory tasks: Brazilian Brief Neuropsychological Assessment Battery (NEUPSILINInf)	(WM deficits: d = 0.49 to 0.96 Episodic memory deficits: d = 0.56 to 0.77)

**Legend:** *SAD, Social Anxiety Disorder; PD, Panic Disorder; GAD, Generalized Anxiety Disorder; OAD, Other Anxiety Disorders; SeAD, Separation Anxiety Disorder; SP, Specific Phobia; OCD, Obsessive–Compulsive Disorder; HC, healthy controls; WM, working memory; STM, short-term memory; LTM, long-term memory; SM, short-term memory; RCFT, Rey–Osterrieth Complex Figure Test; CBTT, Corsi Block-Tapping Test; CVLT, California Verbal Learning Test; NAART, North American Adult Reading Test; ROCF, Rey–Osterrieth Complex Figure Test; NEUPSILIN-Inf, Brazilian Brief Neuropsychological Assessment Battery (Infant version); SSRI, selective serotonin reuptake inhibitors; F, F statistic; t, Student’s t-test; d, Cohen’s d; η^2^, eta squared; p, probability value (two-tailed).*

**Table 4:** Summary of relevant studies investigating memory performance in OCD.

*OCD*			
Shin et al., 2004 [204]	Case–control; 30 OCD vs. 30 HC	ROCF	Markedly ↓ performance on immediate and delayed recall in OCD group
Sawamura et al., 2005 [203]	Case–control; 16 OCD vs. 16 HC	Word categorization, recall, recognition	↓ semantic categorization and recall/recognition accuracy under time constraints
Yue et al., 2021 [211]	Case–control; 55 drug-naive OCD vs. 55 HC	DST, VSMT, SCWT	↓ across working memory components and reduced executive control

Arrows indicate directionality of findings: ↓ = decreased activation/levels. ***Legend.** ROCF, Rey–Osterrieth Complex Figure; DST, Digit Span Test; VSMT, Visuospatial Memory Test; SCWT, Stroop Color–Word Test; OCD, Obsessive–Compulsive Disorder; HC, Healthy Controls.*

**Table 5:** Summary of relevant studies investigating memory performance in psychotic disorders.

*Psychotic Disorder*			
	*Type of Study*	*Tasks*	*Results*
Ibáñez-Casas et al., 2013 [295]	Case–control; N = 429 (DD = 86; HC = 343)	TAVEC	↓ Immediate Recall T1 (d = 0.82); ↓ Learning (Trials 1 & 5, Total Words); ↓ Short/Long Free Recall; ↓ Hits; ↑ Intrusions (d = 0.41); ↑ False Positives (d = 0.54)
Dong et al., 2023 [275]	Cross-sectional; N = 186 (CHR = 42; GHR = 26; FES = 56; HC = 62)	MCCB	↓ Processing Speed, WM, Verbal Learning, Reasoning, Social Cognition (FES vs. HC d = 0.71–1.71; CHR vs. HC d = 0.47–1.46; GHR vs. HC d = 0.36–1.80). Impairments stronger in FES vs. CHR (*p* = 0.008– < 0.001; d = 0.57–1.00) and vs. GHR (*p* = 0.004–0.04; d = 0.73–1.09)
Anticevic et al., 2013 [297]	Cross-sectional, resting-state fMRI; N = 119 (BD = 68, *p* = 34; HC = 51)	Resting-state fMRI at 3T	mPFC dysconnectivity ↓ Amygdala–mPFC ↑ Amygdala–dlPFC ↓ Psychosis severity ↑

Arrows indicate directionality of findings: ↑ = increased activation/levels; ↓ = decreased activation/levels. ***Legend.*** *DD, depressive disorder; HC, healthy controls; CHR, clinical high risk; GHR, genetic high risk; FES, first-episode schizophrenia; p, psychotic features; BD, bipolar disorder; TAVEC, Test de Aprendizaje Verbal España–Complutense (Spanish Verbal Learning Test); MCCB, MATRICS Consensus Cognitive Battery; mPFC, medial prefrontal cortex; dlPFC, dorsolateral prefrontal cortex.*

**Table 6:** Summary of relevant studies investigating memory performance in PTSD.

*PTSD*			
	*Type of Study*	*Tasks*	*Results*
Swick et al., 2017 [236]	Cross-sectional (N = 58; PTSD = 29)	Verbal and visuospatial working tasks	↓ accuracy (Recency effect: F(1,56) = 107.73, *p* < 0.0001, ηp^2^ = 0.658), with no difference in reaction times (F(1,56) = 0.08, *p* = 0.78, ηp^2^ = 0.001). WM impairments correlated with intrusion and hyperarousal symptoms
Petzold & Bunzeck, 2022 [235]	Meta-analysis (N = 3062)	Episodic memory tests (verbal and non-verbal)	↓ episodic memory (d* = −0.50, *p* < 0.0001), with stronger deficits in verbal memory (d* = −0.47 vs. −0.40 non-verbal)
Aupperle et al., 2012 [242]	fMRI (N = 71; PTSD: 37; HC: 34)	fMRI during anticipation of negative/positive emotional images; Neuropsychological tests: WAIS-III Digit Symbol Test, Delis-Kaplan Executive Function System Color-Word Interference Test; Wisconsin Card Sorting Test	↑ DLPFC, ventrolateral PFC/ACC, inferior parietal, precentral; ↓ medial PFC, parahippocampal/amygdala during WM; supports link between poor executive control and intrusive memories
Jelinek et al., 2009 [253]	Comparative Study N = 111 (PTSD = 26; no PTSD = 55; HC = 30)	Autobiographical Memory Task Trauma	memories were significantly more disorganized in the PTSD group (F = 3.16, *p* = 0.05; *t* = 2.48, *p* = 0.02), whereas unpleasant-event memories showed no group differences (*p* > 0.10)

Arrows indicate directionality of findings: ↑ = increased activation/levels; ↓ = decreased activation/levels. ***Legend.*** *PTSD, post-traumatic stress disorder; HC, healthy controls; WM, working memory; PFC, prefrontal cortex; DLPFC, dorsolateral prefrontal cortex; ACC, anterior cingulate cortex; fMRI, functional magnetic resonance imaging; WAIS-III, Wechsler Adult Intelligence Scale—Third Edition.*
